# Blood Glutamate Levels Are Closely Related to Acute Lung Injury and Prognosis after Stroke

**DOI:** 10.3389/fneur.2017.00755

**Published:** 2018-01-19

**Authors:** Wei Bai, Wei Li, Ya-Lei Ning, Ping Li, Yan Zhao, Nan Yang, Yu-Lin Jiang, Ze-Ping Liang, Dong-Po Jiang, Ying Wang, Meng Zhang, Yuan-Guo Zhou

**Affiliations:** ^1^Molecular Biology Center, State Key Laboratory of Trauma, Burn, and Combined Injury, Research Institute of Surgery and Daping Hospital, Third Military Medical University, Chongqing, China; ^2^Department of Neurology, Research Institute of Surgery and Daping Hospital, Third Military Medical University, Chongqing, China; ^3^Department of ICU, Research Institute of Surgery and Daping Hospital, Third Military Medical University, Chongqing, China

**Keywords:** stroke, acute lung injury, blood glutamate, predictor, prognosis

## Abstract

**Background:**

Acute lung injury (ALI) is a serious complication of stroke that occurs with a high incidence. Our preclinical results indicated that ALI might be related to blood glutamate levels after brain injury. The purpose of this study was to assess dynamic changes in blood glutamate levels in patients with stroke and to determine the correlation between blood glutamate levels, ALI, and long-term prognosis after stroke.

**Methods:**

Venous blood samples were collected from controls and patients with stroke at admission and on the third and seventh day after the onset of stroke. Patients were followed for 3 months. The correlations among blood glutamate levels, severities of stroke and ALI, and long-term outcomes were analyzed, and the predictive values of blood glutamate levels and severity scores for ALI were assessed.

**Results:**

In this study, a total of 384 patients with stroke were enrolled, with a median age of 59 years. Patients showed significantly increased blood glutamate levels within 7 days of stroke onset (*p* < 0.05), and patients with more severe injuries showed higher blood glutamate levels. Moreover, blood glutamate levels were closely related to the occurrence (adjusted odds ratio, 3.022, *p* = 0.003) and severity (*p* < 0.001) of ALI and the long-term prognosis after stroke (*p* < 0.05), and they were a more accurate predictor of ALI than the more commonly used severity scores (*p* < 0.01).

**Conclusion:**

These results indicated that an increased blood glutamate level was closely related to the development of ALI and a poor prognosis after stroke.

**Clinical Trial Registration:**

http://www.chictr.org.cn, identifier ChiCTR-RPC-15006770.

## Introduction

With a reported incidence of 20–25% and a high rate of mortality or development of a persistent vegetative state, neurogenic acute lung injury (ALI) is one of the most serious systematic complications following brain injury (1, 2). ALI is primarily characterized by rapidly progressing hypoxemia and pulmonary edema early after brain injury (3, 4). To date, several risk factors for developing ALI after brain injury have been identified, including intracranial (e.g., midline shift and increased intracranial pressure) and extracranial (e.g., administration of vasoactive drugs and a history of drug abuse) factors; however, these factors have either been non-specific or difficult to measure, and an accurate, simple, and stable marker for ALI following brain injury is urgently needed (2, 3, 5).

Stroke, including primarily acute ischemic stroke (AIS), intracerebral hemorrhage (ICH), and spontaneous subarachnoid hemorrhage (SAH), is a serious threat to human health and life (6). After the occurrence of a stroke, elevated glutamate-induced excitotoxicity in the brain is known to severely affect patient prognosis (7, 8). Nevertheless, the role of glutamate levels in the blood has not been well characterized. Glutamate is a non-essential amino acid that participates in a series of basic metabolic reactions, such as deamination and gluconeogenesis (9). In addition, under physiological conditions, the blood levels of glutamate remain relatively stable, and a normal diet prevents significant fluctuations in blood glutamate levels (10); however, certain diets [e.g., rich in monosodium glutamate (MSG)] will cause a significant increase in blood glutamate levels (11). Moreover, researchers have recently found that blood glutamate levels are greatly increased in many patients who experience stroke (12, 13). Our previous animal experiments have indicated that elevated blood glutamate levels might be related to the development of ALI after brain injury (14), although there was no evidence of a close relationship in a large scale clinical study in patients with stroke. Furthermore, since these subtypes of stroke were very different in terms of their pathophysiology, evaluation of whether this is a shared phenomenon between ischemic and hemorrhagic stroke is necessary.

The aim of this study was to evaluate the relationship between blood glutamate levels and the incidence of ALI in patients with stroke, the ability of blood glutamate levels to predict the occurrence of ALI and the relationship between glutamate levels and the long-term prognosis following stroke. The results of this study increase our understanding of this common complication following brain insult.

## Patients and Methods

### Patients

Hospitalized patients were consecutively enrolled from Daping Hospital (Chongqing, China), which has over 2,500 beds and is ranked among the top 100 hospitals in China. It is also a teaching hospital affiliated with the Third Military Medical University and serves as a base for stroke screening and prevention in China. We enrolled patients admitted to the hospital within 6 h of presenting with clinical stroke symptoms and who received a diagnosis of ischemic or hemorrhagic stroke. A control group included 120 subjects who received a routine physical examination, were not diagnosed with stroke, and had no history of stroke/transient ischemic attack (TIA), hypertension, hyperlipidemia, hypercholesterolemia, diabetes mellitus, myocardial infarction, or atrial fibrillation. All participants were Han Chinese living in Chongqing and the surrounding provinces with an age ≥18 years. The diagnosis of stroke was confirmed in all patients by computed tomography (CT) or magnetic resonance imaging. The key exclusion criteria were as follows: transferred from other hospitals or departments; the presence of severe hepatic disease [e.g., aspirate transaminase (AST) levels >40 U/L or alanine aminotransferase (ALT) levels >40 U/L], current or previous lung disease (e.g., tuberculosis, pneumonia, or chronic obstructive pulmonary disease), or abnormal clinical manifestations (e.g., dyspnea, shortness of breath, or cyanosis), and failure of one or more organs. Stroke severity was quantified by a neurologist according to the National Institutes of Health Stroke Scale (NIHSS) score, ICH score, or World Federation of Neurosurgical Surgeons (WFNS) scale at admission and after treatment for patients with AIS, ICH, or SAH, respectively; based on the scores at admission, the patients were divided into mild (NIHSS score: ≤8; ICH score: ≤2; and WFNS scale: I), moderate (NIHSS score: 9–15; ICH score: 3–4; and WFNS scale: II–III), and severe (NIHSS score: ≥16; ICH score: 5–6; and WFNS scale: IV–V) groups (15–18). The detailed sociodemographic and baseline characteristics of patients and controls are listed in Table 1.

**Table 1:** The sociodemographic and baseline characteristics of stroke patients (*n* = 384) and the controls (*n* = 120).

Variables (units)	AIS (*n* = 132)	ICH (*n* = 124)	SAH (*n* = 128)	Control (*n* = 120)	*p*
Age (years)	64 (57–77)	52 (44–61)	58 (46–67)	50 (45–68)	0.075
Female, *n* (%)	59 (44.7)	55 (44.4)	60 (46.9)	70 (58.3)	0.094
Severity scores^a^	15 (9–20)	4 (2–6)	III (II–V)	–	–
Severity scores^d^	10 (5–14)	3 (2–5)	II (I–V)	–	–
Current smoker, *n* (%)	24 (18.2)	19 (15.3)	32 (25.0)	28 (23.3)	0.198
MSG preference, *n* (%)	31 (23.5)	28 (22.6)	26 (20.3)	21 (17.5)	0.660
BMI (kg/m^2^)	24.9 (23.1–26.9)	24.2 (23.3–27.1)	24.5 (22.9–26.9)	24.7 (23.0–26.5)	0.415
OTT (h)	5.0 (1.0–6.0)	4.5 (2.0–5.0)	3.5 (1.0–5.0)	–	0.070

**History, *n* (%)**					
Stroke/TIA	20 (15.2)	28 (22.6)	20 (15.6)	–	0.257
Hypertension	52 (39.4)	64 (51.6)	48 (37.5)	–	0.076
Hyperlipidemia	22 (16.7)	24 (19.4)	20 (15.6)	–	0.721
Hypercholesterolemia	18 (13.6)	20 (16.1)	25 (19.5)	–	0.437
Diabetes mellitus	26 (19.7)	30 (24.2)	32 (25.0)	–	0.598
Myocardial infarction	16 (12.1)	12 (9.7)	16 (12.5)	–	0.776
Atrial fibrillation	20 (15.2)	12 (9.7)	8 (6.3)	–	0.141
Laboratory test^b^					
AST (U/L)	27.0 (21.4–33.3)	28.6 (24.8–38.5)	31.6 (12.9–34.5)	27.2 (15.3–39.0)	0.636
ALT (U/L)	29.7 (21.0–38.7)	28.0 (23.4–32.5)	25.4 (15.3–33.8)	26.1 (19.8–34.1)	0.380

**Complication, *n* (%)**					
Chest infection	16 (12.1)	6 (4.8)	8 (6.3)	–	0.085
ALI	29 (22.0)	15 (12.1)	16 (12.5)	–	0.046
GIH	4 (3.0)	6 (4.8)	6 (4.7)	–	0.721
IH	12 (9.1)	15 (12.1)	11 (8.6)	–	0.603
Epilepsy	7 (5.3)	6 (4.8)	7 (5.4)	–	0.973

**Treatment, *n* (%)**					
IVT	94 (71.2)	–	–	–	–
ET	75 (56.8)	–	–	–	–
Surgical treatment	4 (3.0)	68 (54.8)	100 (78.1)	–	1.12E−05
Ventilation	43 (32.6)	30 (24.2)	29 (22.7)	–	0.071
Length of stay (days)	17 (10–31)	16 (13–26)	10 (7–15)	–	0.001
Hospital mortality, *n* (%)^c^	12 (9.1)	6 (4.8)	8 (6.3)	–	0.396

*Data are presented as the number (%) or median (IQR)*.

*p Values represent differences among the three or four groups using one-way ANOVA (on ranks), χ^2^ test or Fisher’s exact test, as appropriate*.

*AIS, acute ischemic stroke; ALI, acute lung injury; ALT, alanine aminotransferase; AST, aspirate transaminase; BMI, body mass index; ET, endovascular treatment; GIH: gastrointestinal hemorrhage; ICH, intracerebral hemorrhage; IH: intracranial hypertension; IVT, intravenous thrombolysis; MSG, monosodium glutamate; OTT, symptom onset to treatment time; SAH, spontaneous subarachnoid hemorrhage; TIA, transient ischemic attack; IQR, interquartile range; NIHSS, National Institutes of Health Stroke Scale; WFNS, World Federation of Neurosurgical Surgeons*.

*^a^Represents the severity scores at admission*.

*^b^Represents the values at admission*.

*^c^Represents the mortality rate during the hospitalization*.

*^d^Represents the severity scores after treatment. NIHSS score, ICH score, or WFNS scale for patients with AIS, ICH, or SAH, respectively*.

This study was performed in accordance with the recommendations of international ethical guidelines for biomedical research involving human subjects, and the ethics committee of the Research Institute of Surgery and Daping Hospital approved this study (No. 2015-12). All participants (or legal guardians) in this study provided written informed consent in accordance with the Declaration of Helsinki and its later amendments. This clinical trial was registered at the Chinese Clinical Trial Registry (www.chictr.org.cn; unique identifier: ChiCTR-RPC-15006770).

### Diagnosis and Classification of ALI

According to the Berlin Definition for ALI/ARDS (19), after admission, stroke patients with ALI would quickly present (usually within 72 h) with new or worsening respiratory symptoms (e.g., shortness of breath and dyspnea), an acute onset of hypoxemia (PaO_2_/FiO_2_ ≤ 300 mm Hg), and a radiographic examination (representative images, e.g., chest X-ray or CT, are shown in Figure S1 in Supplementary Material) indicating high-density patchy shadows or edema not fully explained by effusion, lobar/lung collapse, or nodules; the respiratory failure symptoms could not be fully explained by cardiac failure, which was excluded by echocardiographic measurements (20, 21). In addition, based on the degree of hypoxemia, the severity of ALI could be classified as mild (200 mm Hg < PaO_2_/FiO_2_ ≤ 300 mm Hg), moderate (100 mm Hg < PaO_2_/FiO_2_ ≤ 200 mm Hg), or severe (PaO_2_/FiO_2_ ≤ 100 mm Hg) (19, 20).

### Clinical Management of Patients with Stroke

Treatment protocols were implemented by a single attending neurologist. For patients with AIS, intravenous thrombolysis (IVT) or endovascular treatment (ET) was performed after rigorous assessment, while for patients with ICH or SAH, surgical (decompressive craniectomy) or conservative treatment (e.g., reduction of intracranial pressure *via* mannitol) was used accordingly. In addition, at the moment the diagnosis of ALI was made, the patients were transferred to the ICU, and treatment was based primarily on ventilation with positive end-expiratory pressure (PEEP), following the ARDSnet protocol (22, 23).

### Blood Collection, Processing, and Glutamate Assay

Venous blood samples were collected from the patients at the time of admission (without any treatment and within 6 h from stroke onset) and on the mornings of the third day and seventh day. Blood was obtained from controls during routine physical examination. All samples were anticoagulated with heparin; then, using standard clinical laboratory methods, the samples were evaluated for inflammatory markers, such as IL-6, C-reactive protein, and procalcitonin, and routine markers, including hepatic function markers, such as ALT and AST, and nerve injury markers, such as neuron-specific enolase (NSE) and S-100B. Blood glutamate levels were assayed using high-performance liquid chromatography with spectrofluorometric detection (Beckman Coulter, CA, USA) at a fixed excitation wavelength of 330 nm and a fixed emission wavelength of 450 nm, according to our previously published methodology (24).

### Outcome Measurement

Mortality data were collected over a follow-up period of 3 months. The long-term outcomes were evaluated by modified Rankin scale (mRS) at 90 days by phone interview by an experienced neurologist. Favorable outcomes and functional independence were defined as an mRS score of 0–2 (25).

### Statistical Analysis

The justification of sample sizes in each group was performed as previously described (26), in which α = 0.05, 1 − β (power analysis) = 0.90, and σ = 10.0. All results are expressed as percentages for categorical variables and as the means (SE or SD) or medians [interquartile range (IQR)] for all variables. Proportions were compared using the χ^2^ test or Fisher’s exact test, and ANOVA followed by Bonferroni *post hoc* tests or Kruskal–Wallis *H* tests, two-tailed *t*-tests, or non-parametric Mann–Whitney *U* tests were used to compare continuous or discrete variables among groups, as appropriate. Spearman correlation or uni-/multivariate logistic regression was used to analyze relationships between blood glutamate levels and other variables [including the occurrence and severity of ALI and clinical characteristics, e.g., age, sex, body mass index (BMI), symptom onset to treatment time (OTT), smoking status, MSG dietary preference, medical history, treatment, and levels of inflammatory markers]. The results are expressed as *r* values or as unadjusted/adjusted odds ratios (ORs) with corresponding 95% confidence intervals (CIs), with *post hoc* power analyses >0.90 (27). Areas under receiver-operating characteristic curves were utilized to evaluate and compare the accuracy of blood glutamate levels and severity scores (NIHSS score, ICH score, and WFNS scale) for predicting the occurrence of ALI (28). In addition, the possibility of defining a “cutoff value” of blood glutamate level associated with survival and poor functional outcome was explored using Kaplan–Meier survival curve analysis, followed by Log Rank testing and multivariate logistic regression, respectively. Two-tailed *p* values <0.05 represent statistically significant differences. All statistical analyses were performed using Sigma Plot (version 12.5; Systat Software Inc., San Jose, CA, USA).

## Results

**Figure 1:**
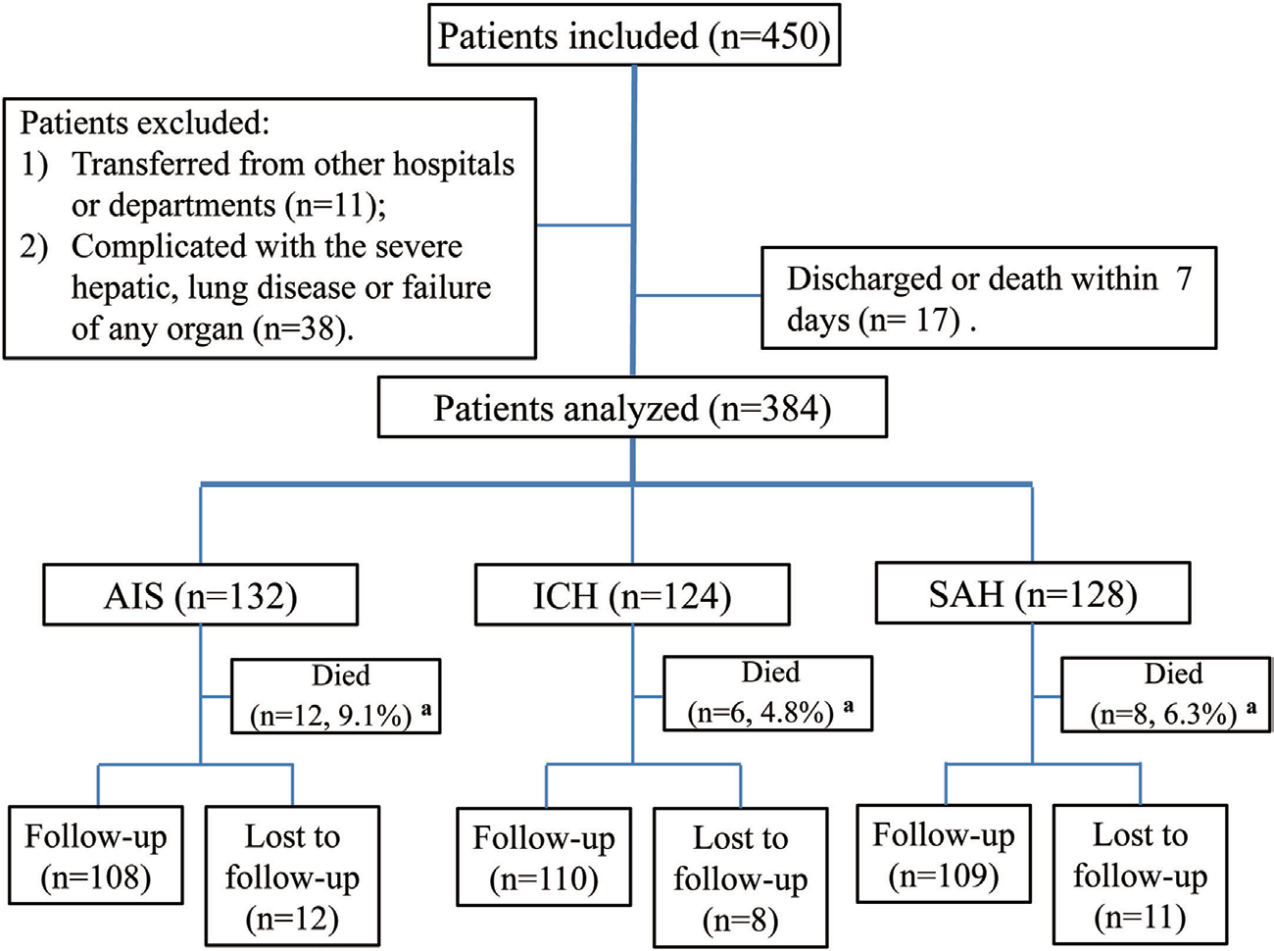
Study flowchart. Abbreviations: AIS, acute ischemic stroke; ICH, intracerebral hemorrhage; SAH, spontaneous subarachnoid hemorrhage. ^a^Represents death during hospitalization.

Between August 2015 and July 2016, a total of 450 patients were consecutively screened in our institution’s Department of Neurology and Department of ICU. Of these, 49 patients were excluded, and 17 patients discharged or died within 7 days. Ultimately, 384 patients were included and analyzed in this study, including 132 patients with AIS, 124 patients with ICH, and 128 patients with SAH (Figure 1). The median age of the study participants was 59 years (range, 18–89), the median NIHSS score, ICH score, or WFNS scale for patients with AIS, ICH, or SAH at admission was 15, 4, and III, respectively; after treatment, the median NIHSS score decreased markedly (*p* = 0.032). The median time of OTT for all patients was 5 h. There was no significant difference among these three subgroups of patients concerning the number of current smokers and MSG dietary preferences, medical history, and laboratory tests (*p* > 0.05). Thirty patients (7.8%) suffered a chest infection (pneumonia or bronchitis), 60 (15.6%) were diagnosed with ALI, and 16 (4.2%), 38 (9.9%), and 20 (5.2%) patients developed gastrointestinal hemorrhage, intracranial hypertension, and epilepsy, respectively. Ninety-four patients (71.2%) with AIS were treated with IVT, and 75 patients (56.8%) were treated with ET. Surgery was performed on most patients with ICH (54.8%) or SAH (78.1%), and 102 (26.6%) received ventilation therapy (Table 1). Most of the ALI cases (91.6%) developed within 24 h after brain injury, and the prevalence of severe ALI was 31.7%. All patients diagnosed with ALI were managed in the ICU with a median PEEP value of 8 cm H_2_O (Table 2). The total mortality was 6.8% (26 patients, including 12 with ALI) after admission during the hospitalization.

**Table 2:** Clinical characteristics of patients with ALI (*n* = 60).

Variables (units)	Values
Age (years)	64 (43–72)
Gender, female	38 (63.3)
APACHE II score	31 (24–38)
Current smoker, *n* (%)	11 (18.3)
MSG dietary preference, *n* (%)	13 (21.7)
BMI (kg/m^2^)	24.1 (22.3–25.7)
OTT (h)	4.0 (2.0–6.0)
**History, *n* (%)**	
Stroke/TIA	21 (35.0)
Hypertension	43 (71.7)
Hyperlipidemia	25 (41.7)
Hypercholesterolemia	23 (38.3)
Diabetes mellitus	39 (65.0)
Myocardial infarction	21 (35.0)
Atrial fibrillation	19 (31.7)
Arterial blood gas analysis	
PaO_2_ (<60 mm Hg), *n* (%)	33 (55.0)
PaCO_2_ (>50 mm Hg), *n* (%)	19 (31.7)
PH	7.32 (7.24–7.38)
SpO_2_ (%)	91 (86–96)
PaO_2_/FiO_2_	221 (171–260)
**ALI diagnosis after admission (h), *n* (%)**	
<12	35 (58.3)
12–24	20 (33.3)
24–36	5 (8.4)
**ALI severity, *n* (%)**	
Mild	24 (40.0)
Moderate	17 (28.3)
Severe	19 (31.7)
**Treatment**	
IVT or ET, *n* (%)	20 (33.3)
Surgical treatment, *n* (%)	31 (51.7)
PEEP (cm H_2_O)	8 (5–12)
Length of ventilation (days)	5 (3–11)
Length of hospital stay (days)	11 (8)
Death in hospital, *n* (%)^a^	6 (10.0)

*Data are presented as the number (%), median (IQR), or mean (SD)*.

*ALI, acute lung injury; APACHE, acute physiology and chronic health evaluation; BMI, body mass index; ET, endovascular treatment; IVT, intravenous thrombolysis; MSG, monosodium glutamate; OTT, symptom onset to treatment time; PEEP, positive end-expiratory pressure; TIA, transient ischemic attack; IQR, interquartile range*.

*^a^Represents the mortality rate during hospitalization*.

### Alterations in Blood Glutamate Levels in Patients with Different Causes and Severities of Stroke

**Figure 2:**
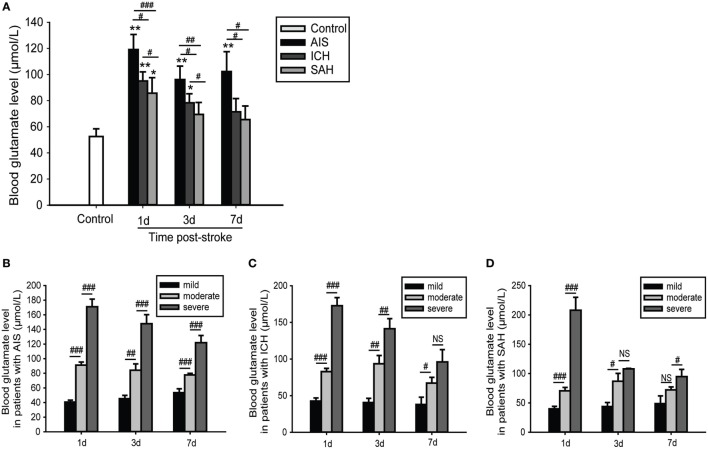
Time- and severity-dependent changes in blood glutamate levels in patients with stroke. **(A)** Comparison of blood glutamate levels with different causes of stroke. **(B–D)** Comparison of blood glutamate levels with different severities of stroke. *p < 0.05, **p < 0.01, and ***p < 0.001 compared with the control group; ^#^*p* < 0.05, ^##^*p* < 0.01, and ^###^*p* < 0.001 compared between the two groups. Data are expressed as the means (SE), and the significance was determined by ANOVA followed by Bonferroni *post hoc* tests or Kruskal–Wallis *H* tests. Abbreviations: AIS, acute ischemic stroke; ICH, intracerebral hemorrhage; NIHSS, National Institutes of Health Stroke Scale; SAH, spontaneous subarachnoid hemorrhage.

The observed alterations in blood glutamate levels varied among patients with different causes of stroke; glutamate levels were highest and most sustained in patients with AIS, followed by patients with ICH and those with SAH. However, for each subgroup of stroke, there were relatively few changes in glutamate levels within 7 days of admission (Figure 2A). We further found that patients with severe or moderate stroke exhibited significantly increased blood glutamate levels; these levels were higher with increased stroke severity (Figures 2B–D).

### Blood Glutamate Levels but Not Severity Scores Were Closely Related to the Incidence and Severity of ALI

**Figure 3:**
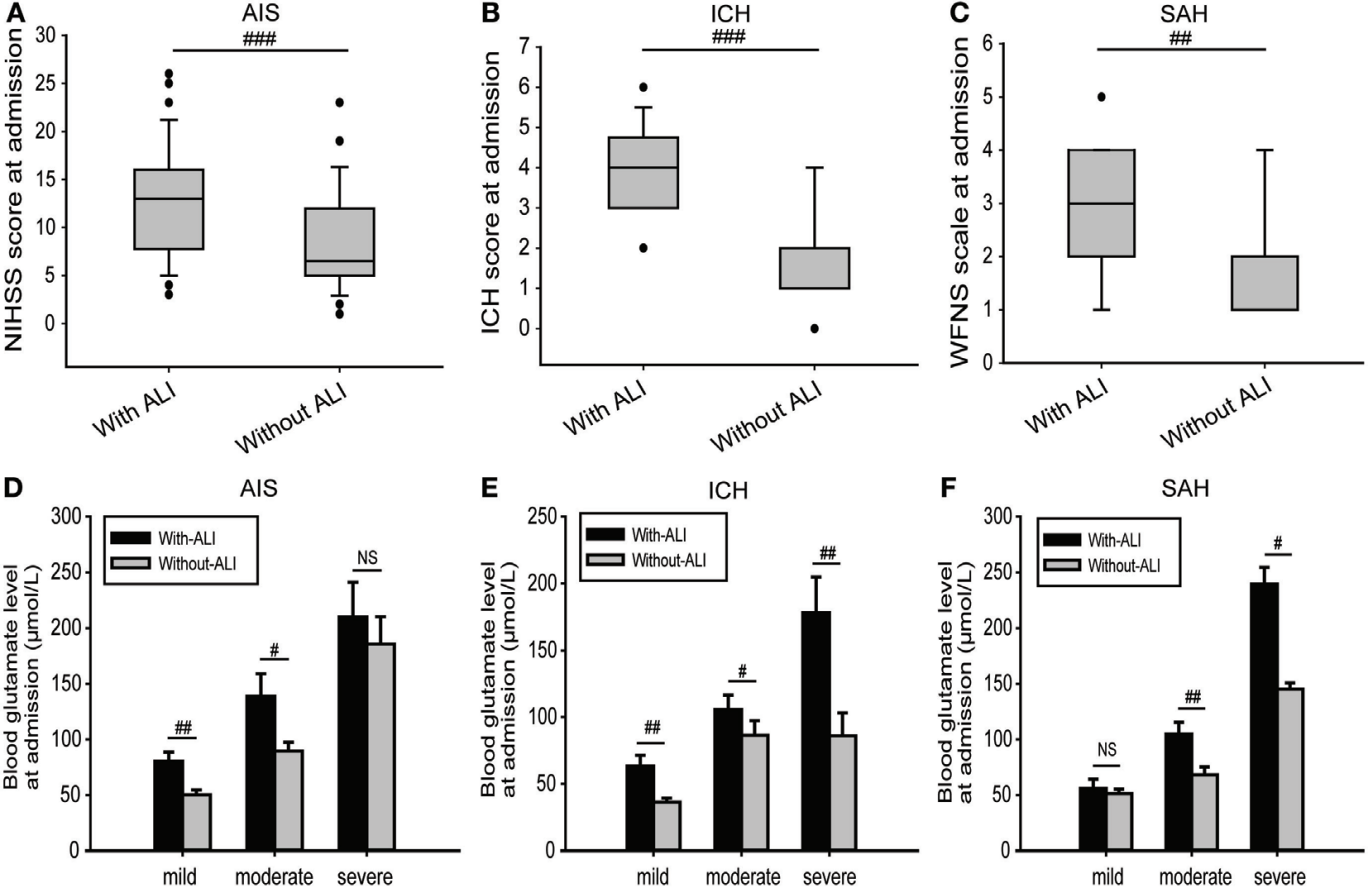
Comparisons of severity scores and blood glutamate levels at admission between patients with and those without ALI. **(A–C)** Comparison of severity scores at admission for patients in different subgroups and with or without ALI. **(D–F)** Comparison of blood glutamate levels at admission between different severities in patients with AIS, ICH, or SAH. Data are expressed as the median (IQR) **(A–C)** and means (SE) **(D–F)**. ^#^*p* < 0.05, ^##^*p* < 0.01, and ^###^*p* < 0.001 by two-tailed Student’s *t*-tests or non-parametric Mann–Whitney *U* tests; NS, no significance. Abbreviations: AIS, acute ischemic stroke; ICH, intracerebral hemorrhage; NIHSS, National Institutes of Health Stroke Scale; SAH, spontaneous subarachnoid hemorrhage; WFNS, World Federation of Neurosurgical Surgeons; ALI, acute lung injury; IQR, interquartile range.

For all of the stroke patients, the median severity scores of patients who developed ALI were much higher than those without ALI (Figures 3A–C). However, logistic regression analysis showed that these were not closely related with the occurrence of ALI when adjusted for confounding factors (adjusted OR: 1.060, 95% CI: 0.852–1.320, *p* = 0.600 for NIHSS score; adjusted OR: 1.083, 95% CI: 0.890–1.161, *p* = 0.060 for ICH score; and adjusted OR: 1.036, 95% CI: 0.974–1.390, *p* = 0.153 for WFNS scale) (Table 3). However, blood glutamate levels were much higher in patients with ALI with different stroke severities (Figures 3D–F), and the increased blood glutamate levels were associated with a higher risk of ALI (adjusted OR: 3.022, 95% CI: 2.001–4.043, *p* = 0.003) (Table 3). Moreover, the blood glutamate levels were closely related to the severity of ALI in patients with AIS (*r* = 0.591, *p* = 1.198E−04), ICH (*r* = 0.564, *p* = 2.627E−05), and SAH (*r* = 0.842, *p* = 2.922E−04).

**Table 3:** ALI outcomes in relation to baseline and treatment characteristics of patients with stroke.

Variables	Unadjusted OR (95% CI)	*p*	Adjusted OR (95% CI)^a^	*p*
Age >60 years	0.762 (0.408–1.423)	0.394	–	–
Female sex	0.793 (0.424–1.483)	0.467	–	–
BMI (kg/m^2^)	1.301 (0.660–2.563)	0.447	–	–
OTT (h)	0.984 (0.496–1.954)	0.963	–	–
Current smoker	1.280 (0.960–1.651)	0.200	–	–
MSG preference	0.828 (0.721–1.093)	0.072	–	–

**Medical history**				
Stroke/TIA	0.942 (0.237–3.812)	0.950	–	–
Hypertension	0.778 (0.458–0.992)	0.450	–	–
Hyperlipidemia	0.955 (0.529–1.270)	0.080	–	–
Hypercholesterolemia	0.904 (0.484–1.320)	0.502	–	–
Diabetes mellitus	0.694 (0.364–1.289)	0.910	–	–
Myocardial infarction	0.978 (0.956–1.000)	0.050	–	–
Atrial fibrillation	0.982 (0.742–1.299)	0.899	–	–

**Treatment**				
IVT or ET	0.879 (0.272–2.838)	0.829	–	–
Surgical treatment	0.797 (0.217–2.929)	0.733	–	–
Ventilation	0.903 (0.236–3.459)	0.882	–	–

**Inflammatory markers**				
IL-6	1.001 (0.992–1.010)	0.827	1.194 (0.806–1.364)	0.075
CRP	1.050 (0.981–1.016)	0.118	1.314 (0.620–1.603)	0.096
PCT	0.967 (0.895–1.045)	0.392	1.156 (0.844–1.133)	0.065
WBCs	1.128 (0.683–1.862)	0.638	1.172 (0.828–1.384)	0.201
Neutrophils	1.030 (0.599–1.770)	0.915	0.805 (0.650–1.641)	0.280

NIHSS score^b^	1.311 (1.057–1.625)	0.014	1.060 (0.852–1.320)	0.600
ICH score^b^	2.810 (1.808–4.368)	1.823E−04	1.083 (0.890–1.161)	0.060
WFNS scale^b^	1.933 (1.412–2.509)	0.021	1.036 (0.974–1.390)	0.153
Blood glutamate^c^	4.837 (2.731–8.715)	7.659E−04	3.022 (2.001–4.043)	0.003

*BMI, body mass index; CRP, C-reactive protein; ET, endovascular treatment; IVT, intravenous thrombolysis; MSG, monosodium glutamate; OTT, symptom onset to treatment time; PCT, procalcitonin; TIA, transient ischemic attack; WBCs, white blood cells; ALI, acute lung injury; AIS, acute ischemic stroke; CI, confidence interval; ICH, intracerebral hemorrhage; OR, odds ratio, WFNS, World Federation of Neurosurgical Surgeons*.

*^a^Adjusted for age, sex, BMI, OTT, current smoker, MSG preference, medical history, treatment, and inflammatory markers*.

*^b^NIHSS score, ICH score, or WFNS scale at admission was analyzed in patients with AIS, ICH, or SAH, respectively*.

*^c^Represents blood glutamate level at admission*.

### Blood Glutamate Level Analysis As a Sensitive and Specific Method for Predicting ALI

**Figure 4:**
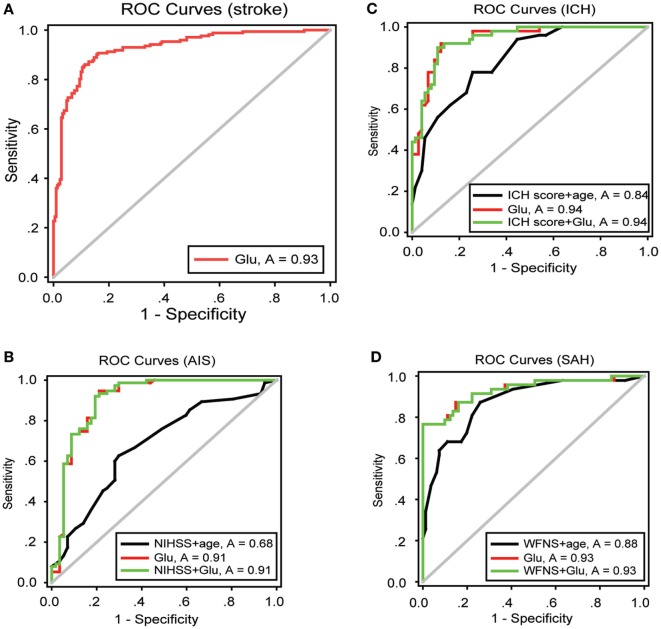
ROC curves showing the sensitivity versus specificity of different models for predicting ALI. **(A)** For all patients with stroke, the area under the curve was 0.931 (95% CI: 0.905–0.957) for glutamate levels at admission. **(B–D)** ROC curves for severity scores combined with age, glutamate levels at admission, and severity scores combined with glutamate levels in patients with AIS, ICH, and SAH, respectively. Abbreviations: AIS, acute ischemic stroke; ICH, intracerebral hemorrhage; NIHSS, National Institutes of Health Stroke Scale; SAH, spontaneous subarachnoid hemorrhage; WFNS, World Federation of Neurosurgical Surgeons; CI, confidence interval; ROC, receiver-operating characteristic.

To obtain an early and accurate diagnosis of ALI, we next generated receiver-operating characteristic curves to assess the predictive accuracy of both blood glutamate levels and severity scores at admission. For all the patients with stroke, the optimal cutoff value for blood glutamate as an indicator of ALI was projected to be 87.6 µmol/L, which yielded an area under the curve (AUC) of 0.931 (95% CI: 0.905–0.957), with a sensitivity of 0.878 and a specificity of 0.892 (Figure 4A). The likelihood ratio test showed a significant increase in the predictive value of blood glutamate levels over severity scores (*p* = 1.665E−04, *p* = 0.002, and *p* = 0.009 for patients with AIS, ICH, and SAH, respectively). Moreover, the combination of glutamate levels and severity scores resulted in AUCs that were superior to severity scores but not superior to blood glutamate levels alone for predicting ALI (Figures 4B–D).

### Association between Blood Glutamate Levels and Long-term Clinical Outcomes

**Figure 5:**
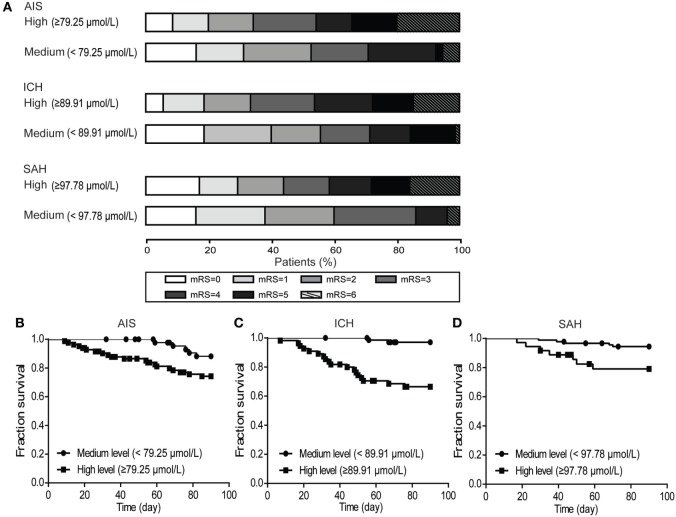
The distribution of modified Rankin scale (mRS) scores at 90 days and the poststroke survival curves. **(A)** Multivariate logistic regression analysis showed that patients with high blood glutamate levels were less likely to develop functional independence in patients with acute ischemic stroke (AIS) (*p* = 0.013), intracerebral hemorrhage (ICH) (*p* = 2.047E−04), and SAH (*p* = 1.684E−04). **(B–D)** Kaplan–Meier survival curve analysis showed that the survival rate was significantly lower in patients with high blood glutamate levels (AIS, *p* = 0.039; ICH, *p* = 1.325E−05; and SAH, *p* = 0.008).

Thirty-one patients (8.7%) were lost to follow-up during the 90 days following discharge from the hospital. The analysis of blood glutamate level cutoff suggested that the survival rate was significantly lower for patients with high blood glutamate levels (AIS, *p* = 0.039; ICH, *p* = 1.325E−05; and SAH, *p* = 0.008) (Figures 5B–D). The distributions of mRS scores at 90 days in patients with the three stroke subtypes are shown in Figure 5A. For all three subtypes, after adjustment for potential confounders, patients with high blood glutamate levels were less likely to report functional independence [AIS (43.9 versus 60.0%; adjusted OR: 1.041, 95% CI: 1.039–1.043, *p* = 0.013), ICH (33.4 versus 55.7%; adjusted OR: 1.024, 95% CI: 1.023–1.025, *p* = 2.047E−04), and SAH (34.3 versus 52.7%; adjusted OR: 1.019, 95% CI: 1.018–1.020, *p* = 1.684E−04)]. In addition, in patients who died within 90 days of follow-up, cases with ALI who were diagnosed in the hospital were more likely to die than those without ALI (*p* = 0.004).

## Discussion

In this preliminary clinical study, we found that patients who experienced stroke (both hemorrhagic and ischemic) exhibited significantly increased blood glutamate levels; these levels could rise for approximately 7 days or more, according to previous work (12). In addition, we found that during hospitalization after a stroke (within 7 days), increased disease severity was associated with higher levels of blood glutamate. Patients with AIS exhibited higher levels of blood glutamate than patients with ICH or SAH; the proportion of patients in the AIS group who were diagnosed with ALI was also much higher than that in the ICH group and the SAH group. We speculate that this was partly because the severity of patients with AIS that we enrolled in this study was higher than that previously reported (12). Patients with ALI showed higher levels of brain injury markers (NSE and S-100B) (Table S1 in Supplementary Material) and severity scores than patients without ALI. Nevertheless, we found that blood glutamate levels but not the severity scores of stroke patients at admission could serve as a risk factor for the development of ALI, which was demonstrated by regression analysis after controlling for many potential confounding factors (including age, sex, BMI, and OTT, current smoking status, MSG dietary preference, medical history, treatment, and inflammatory markers). It was noteworthy that patients with a history of myocardial infarction showed a marginal association (*p* = 0.050) with ALI, partly because these patients always showed other risk factors for stroke (29). The severity scores were always influenced by these confounding factors, especially the OTT (30, 31), which may partly account for these discrepancies.

As the clinical manifestations of ALI lack specificity and are often overshadowed by primary disease, and because ALI typically develops rapidly (32), there is an urgent need to identify an objective and easily measurable parameter that can predict the occurrence of ALI. Several scores at admission have been commonly used to evaluate the severity and predict prognosis after brain injury (33–35); however, we found that blood glutamate levels were more accurate and reliable for predicting the occurrence of ALI and were closely related to the long-term prognosis. In addition, we found blood glutamate levels were always much higher in patients with ALI than in patients without ALI; in patients with ALI, blood glutamate levels gradually decreased over the length of the hospital stay, whereas few changes in blood glutamate levels were observed in patients without ALI (Figure S2B in Supplementary Material). These results collectively demonstrate the potential of using blood glutamate levels in predicting ALI after stroke and indicate that although the pathogenesis is different, the predictive value of blood glutamate levels in different types of stroke is worthy of further investigation.

The inflammatory reaction is an important facet of the occurrence and development of ALI (21). In this study, we also measured the levels of inflammatory markers in blood; we found that patients with ALI had much higher levels of inflammatory markers than patients without ALI (Table S1 in Supplementary Material), and additional analysis showed a positive correlation between blood glutamate levels and IL-6 levels during hospitalization (Figure S3 in Supplementary Material). After brain injury, the activation of the sympathetic nervous system and the hypothalamic–pituitary–adrenal axis has been shown to be associated with the development of systemic inflammatory complications (36, 37); however, it is noteworthy that in patients without ALI but who were diagnosed with a complicated chest infection, even though inflammatory mediator levels in these patients were similar to those in patients with ALI, the mean glutamate levels in these patients were much lower (Figure S2A in Supplementary Material). In addition, our previous animal experiments demonstrated that high concentrations of blood glutamate mediate interactions between the adenosine A_2A_ receptor and the metabotropic glutamate receptor 5 on neutrophils, thereby inducing the release of inflammatory mediators (14). These results indicate not only that the elevated blood glutamate and inflammatory marker levels were not simply a concomitant phenomenon but also that blood glutamate might play a precipitating role in the occurrence of ALI, and this role may be related to the pro-inflammatory effects of glutamate. However, inflammatory cells, including neutrophils (38) and platelets (12, 39), also release glutamate into the blood after brain injury, thereby forming a mutually amplifying system that strengthens the effects of blood glutamate. These hypotheses and the specific mechanism underlying this phenomenon require further validation in animal studies. Nevertheless, as several studies have reported that hemodialysis and peritoneal dialysis are effective ways of lowering blood glutamate levels (40, 41), these might serve as a potential and effective measure for reducing blood glutamate levels to prevent or treat ALI after stroke.

This study has several limitations. First, this was a single-center observational study with a relatively small sample size. Although the interference of several known factors was controlled for statistically based on logistic regression, such as age, sex, BMI, OTT, current smoking status, MSG dietary preference, medical history, treatment, and inflammatory markers, which have been previously shown to be a risk factor for stroke (29), a potential modulating factor for blood glutamate levels (10), or disease progression (42), it is possible that hidden confounding factors exist, and our results should be interpreted carefully. Therefore, additional larger prospective studies would strengthen our conclusions. Moreover, patients were recruited over a relatively long period of time, which may have introduced a diagnostic bias for ALI based on the individual experiences of different clinicians. Finally, on the first day, only one blood sample but multiple severity scores were often obtained; therefore, we analyzed the relationship between these variables only at admission. Besides, in this study, we found acute treatment could obviously alleviate the severity of brain injury (especially in patients with AIS), which indicated the possible effects of acute treatment in the levels of blood glutamate and the occurrence of ALI. However, the analysis of additional blood samples taken at different time points through one-to-one correlation analyses and a strict randomized control trial in a future study was needed to test and confirm these effects.

## Conclusion

This study further confirms the results in our previous animal studies and demonstrates that blood glutamate levels are closely related to the incidence of ALI in three subtypes of stroke patients. Moreover, our findings suggest that high blood glutamate levels are a potential effective predictor for ALI and a risk factor for the development of ALI and poor prognosis after stroke.

## Ethics Statement

This study was performed in accordance with the recommendations of international ethical guidelines for biomedical research involving human subjects, and the ethics committee of the Research Institute of Surgery and Daping Hospital approved this study (No. 2015-12). All participants (or legal guardians) in this study provided written informed consent in accordance with the Declaration of Helsinki and its later amendments.

## Author Contributions

WB, WL, Y-LN, and Y-GZ designed the research and drafted the manuscript. PL and YZ analyzed the data. WB, NY, and Y-LJ performed the experiments. Z-PL, D-PJ, YW, and MZ helped perform the experiments. Y-GZ revised the manuscript and contributed reagents, materials, and analysis tools. All the authors read and approved the final manuscript.

## Conflict of Interest Statement

The authors declare that the research was conducted in the absence of any commercial or financial relationships that could be construed as a potential conflict of interest.

## Abbreviations

AIS, acute ischemic stroke; ALI, acute lung injury; AUC-ROCs, areas under receiver-operating characteristic curves; BMI, body mass index; GIH, gastrointestinal hemorrhage; ICH, intracerebral hemorrhage; IH, intracranial hypertension; MSG, monosodium glutamate; NIHSS, National Institutes of Health Stroke Scale; OTT, symptom onset to treatment time; PEEP, positive end-expiratory pressure; SAH, spontaneous subarachnoid hemorrhage; TIA, transient ischemic attack; WFNS, World Federation of Neurosurgical Surgeons.
