# Supplementary material for: Blood Glutamate Levels Are Closely Related to Acute Lung Injury and Prognosis after Stroke
Source: Front Neurol. 2018 Jan 19;8:755. doi: 10.3389/fneur.2017.00755 (PMC5785722; doi:10.3389/fneur.2017.00755)
Supplement: Supplementary file 1 [file Table_1.PDF]

Supplemental Table 1. Laboratory Test Results from Patients with or without ALI

	Stroke Patients		p
	With ALI	Without ALI	
Inflammatory markers [a]			
IL-6 (ng/L)	11.57 (3.24-27.35)	6.36 (2.67-12.11)	0.041
CRP (mg/L)	16.0 (5.0-36.0)	8.0 (4.6-26.4)	0.039
PCT (μg/L)	1.06 (1.04-1.14)	1.06 (1.04-1.08)	0.064
WBCs (^10^9)	10.91 (8.38-13.54)	10.19 (7.54-12.29)	0.012
Neutrophils (^10^9)	8.59 (6.15-11.42)	8.20 (5.51-10.28)	0.045
Nerve injury markers [a]			
NSE (μg/L)	6. 63 (5.75-9.87)	5.44 (3.66-8.59)	0.024
S-100B (μg/L)	0.59 (0.31-0.73)	0.28 (0.23-0.62)	0.016
Hepatic function [a]			
AST (U/L)	31.5 (22.7-38.6)	29.6 (18.5-33.9)	0.362
ALT (U/L)	26.5 (21.6-39.7)	25.8 (23.8-37.0)	0.867

Data are presented as median (IQR) values. ALI: acute lung injury; ALT, alanine aminotransferase; AST, aspirate transaminase; CRP, C-reactive protein; NSE, neuron-specific enolase; PCT, procalcitonin; WBCs, white blood cells. *P* values represent differences between the groups with or without ALI based on two-tailed Student's t-tests or nonparametric Mann-Whitney U tests.

[a] Represents the values at admission.
