# Supplementary material for: Blood Glutamate Levels Are Closely Related to Acute Lung Injury and Prognosis after Stroke
Source: Front Neurol. 2018 Jan 19;8:755. doi: 10.3389/fneur.2017.00755 (PMC5785722; doi:10.3389/fneur.2017.00755)
Supplement: Supplementary file 2 [file Image_1.PDF]

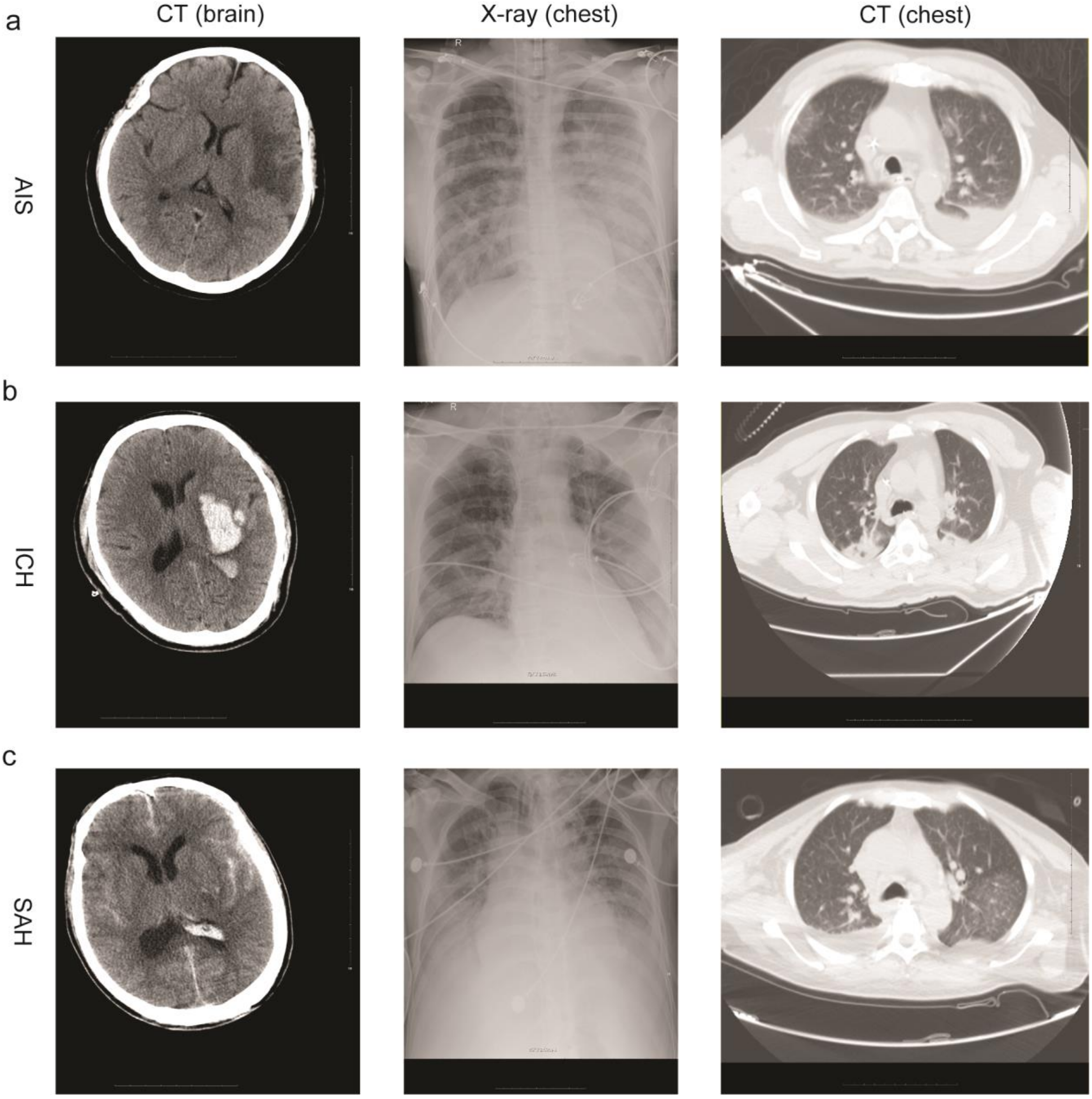

Supplemental Figure 1. Representative radiographic examination images of patients with ALI after stroke.

Representative images of patients with AIS (**a**), ICH (**b**) and SAH (**c**), especially for complicated ALI, are shown. Chest X-rays or CT scans showed marked high-density patchy shadows in both lungs. AIS, acute ischemic stroke; ICH, intracerebral haemorrhage; SAH, spontaneous subarachnoid haemorrhage.
