# Supplementary material for: Blood Glutamate Levels Are Closely Related to Acute Lung Injury and Prognosis after Stroke
Source: Front Neurol. 2018 Jan 19;8:755. doi: 10.3389/fneur.2017.00755 (PMC5785722; doi:10.3389/fneur.2017.00755)
Supplement: Supplementary file 3 [file Image_2.PDF]

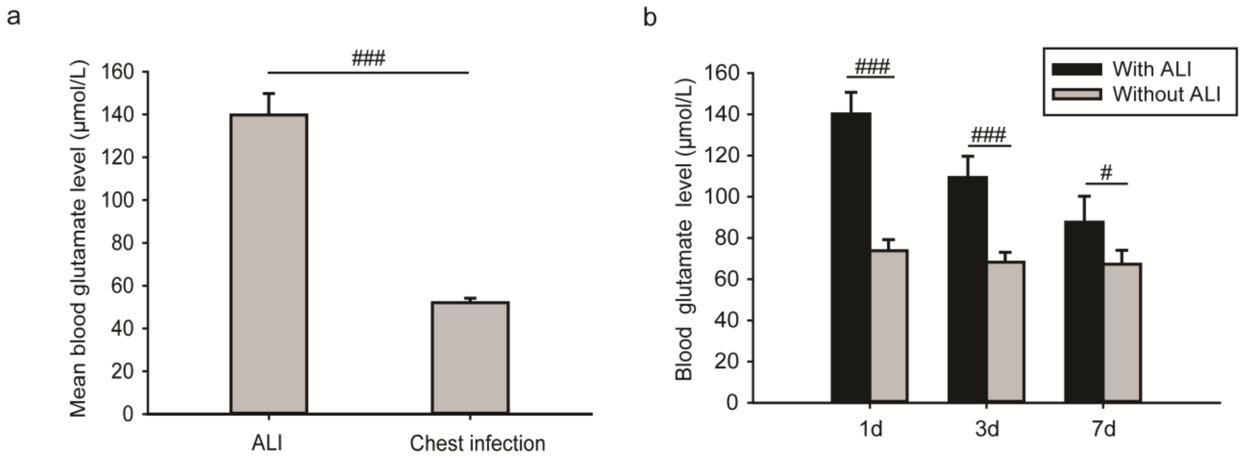

Supplemental Figure 2. Comparisons of blood glutamate levels between patients with/without ALI.

(**a**). Mean blood glutamate levels in patients with ALI or chest infection. $^{###}p<0.001$ between the two groups. (**b**). Blood glutamate levels in all patients with or without ALI on the 1st, 3rd and 7th days after stroke. $^{#}p<0.05$ and $^{###}p<0.001$ between the two groups. Significance was determined by two-tailed Student's t-tests or nonparametric Mann-Whitney U tests. ALI, acute lung injury.
