# Supplementary material for: Blood Glutamate Levels Are Closely Related to Acute Lung Injury and Prognosis after Stroke
Source: Front Neurol. 2018 Jan 19;8:755. doi: 10.3389/fneur.2017.00755 (PMC5785722; doi:10.3389/fneur.2017.00755)
Supplement: Supplementary file 4 [file Image_3.PDF]

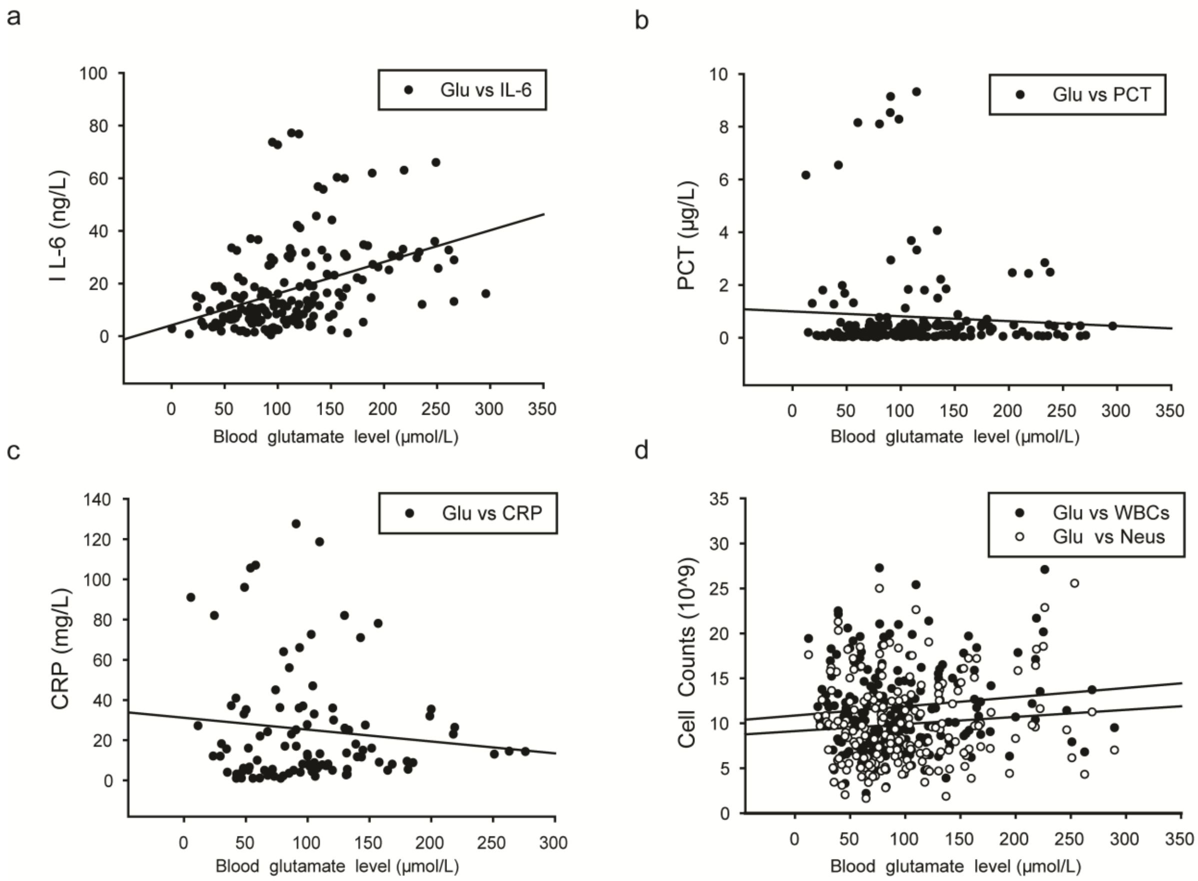

Supplemental Figure 3. Relationships between blood glutamate levels and inflammatory parameters at admission.

For IL-6 (**a**), the correlation was significant (r=0.442, p=0.000); for PCT (**b**), CRP (**c**), WBCs and Neuts (**d**), there were no significant correlations (p>0.05). CRP: C-reactive protein; Glu: glutamate; Neuts: Neutrophils; PCT, procalcitonin; WBCs, white blood cells.
